# Development and Characterization of Kombucha Tea Nanoemulsion for Stability, Bioactive Delivery, and Functional Food Applications

**DOI:** 10.3390/foods15142468

**Published:** 2026-07-12

**Authors:** Thida Kaewkod, Nitsanat Cheepchirasuk, Wipawadee Teppabut, Chayangkorn Kuntolbut, Yingmanee Tragoolpua

**Affiliations:** 1 Department of Biology, Faculty of Science, Chiang Mai University, Chiang Mai 50200, Thailand; thida.kaewkod@cmu.ac.th (T.K.); nitsanat_cheep@cmu.ac.th (N.C.); vipawadee_th@cmu.ac.th (W.T.); chayangkorn_k@cmu.ac.th (C.K.); 2 Natural Extracts and Innovative Products for Alternative Healthcare Research Group, Faculty of Science, Chiang Mai University, Chiang Mai 50200, Thailand

**Keywords:** antioxidant activity, bioactive compounds, cellular uptake, functional food, kombucha tea, nanoemulsion

## Abstract

Kombucha is a fermented tea beverage and a rich source of bioactive compounds with potential health benefits. This study aimed to evaluate the effects of different tea substrates (green, oolong, and black tea) on fermentation characteristics, bioactive compound profiles, antioxidant activity, and cytotoxicity, and to develop a nanoemulsion system for the encapsulation of kombucha-derived bioactive compounds for food applications. Kombucha was fermented for 15 days and during microbial growth, pH, total acidity, and sugar consumption were monitored. The results showed that green tea kombucha exhibited the lowest pH and highest total acidity, indicating more active fermentation. High-performance liquid chromatography analysis revealed that concentrated green tea kombucha contained higher levels of catechin, caffeine, and selected organic acids, whereas black tea kombucha exhibited the highest epigallocatechin gallate (EGCG) concentration. Among the tested samples, green tea kombucha demonstrated superior antioxidant properties as determined by DPPH, ABTS, and FRAP assays, while exhibiting no significant cytotoxicity in RAW 264.7, Caco-2, and A549 cells. Based on these findings, a nanoemulsion system was developed using concentrated green tea kombucha. The nanoemulsion exhibited an average particle size of 110.03 ± 6.2 nm, a polydispersity index of 0.28 ± 0.01, and a zeta potential of +32.5 ± 0.5 mV. Furthermore, the nanoemulsion maintained acceptable physicochemical stability during 6 months of storage at 4 °C, remaining within the nanoscale range with only minor changes in PDI and zeta potential. The encapsulation efficiency reached 91.66 ± 2.29%, and the system demonstrated a biphasic release profile with an initial burst followed by sustained release. Cellular uptake studies confirmed efficient internalization of the nanoemulsion in all tested cell lines. These findings highlight the potential of green tea kombucha as a functional food ingredient and demonstrate the feasibility of nanoemulsion-based systems for the encapsulation and cellular internalization of kombucha-derived bioactive compounds. This study provides a promising strategy for the development of value-added fermented beverages and functional food products.

## 1. Introduction

Kombucha is a fermented tea beverage that has gained increasing global popularity in recent years, particularly among consumers seeking functional foods and beverages with potential health-promoting properties. Kombucha tea is a production from various of tea (*Camellia sinensis*), generally such as green, oolong and black tea. Kombucha is produced through the fermentation of sweetened tea by a symbiotic culture of bacteria and yeast (SCOBY), which consists primarily of acetic acid bacteria, lactic acid bacteria, and yeasts. A characteristic feature of kombucha fermentation is the formation of a cellulose-like pellicle at the air–liquid interface of the fermentation medium [1]. During fermentation, yeasts hydrolyse sucrose to glucose and fructose as a by-product of ethanol fermentation, while acetic acid bacteria then convert ethanol and glucose into acetic acid and gluconic acid, respectively [2]. Other metabolites are produced as a result of kombucha fermentation including other acid such as lactic, citric, malic, glucuronic, ascorbic, succinic, and saccharic acids, vitamins, minerals, and phenolic compounds [3,4]. The biological properties of kombucha have been attributed to the combined effects of tea-derived polyphenols, fermentation-derived organic acids, microbial metabolites, enzymes, and other bioactive compounds generated during fermentation [5,6,7]. However, the content of bioactive compounds varies depending on fermentation conditions, tea type, and the microorganisms involved. Therefore, fermentation kinetics and parameters of the process should be carefully monitored and controlled to ensure consistent product quality. Kombucha tea has recently become more popular beverage, and this popularity is probably due to its health benefits, along with investigations toward the role of the microbiome in human health [8]. Several studies have reported that kombucha exhibits antioxidant and anti-inflammatory properties and may provide other health-promoting effects. However, these biological activities can vary depending on tea substrate, fermentation conditions, and microbial composition [3,9]. Previous studies have also investigated the effects of kombucha extracts on various cancer cell lines, including colorectal, breast, and prostate cancer models [4,10,11,12].

Kombucha is traditionally consumed as a fermented beverage without further modification following fermentation. To enhance the functionality and delivery of kombucha-derived bioactive compounds, nanotechnology-based delivery systems have attracted increasing research interest. Nanoemulsion is a stable dispersion of two immiscible liquids, like oil and water, where one liquid is broken into droplets smaller than 200 nanometers in size [13]. This is achieved by mixing the liquid with surfactant to create sub-micron droplets, which are then stabilized by an interfacial film. Due to their small droplet size and large interfacial area, nanoemulsions may improve the dispersion, physicochemical stability, and controlled release of encapsulated bioactive compounds [14,15]. Previous studies have explored the encapsulation of kombucha-derived bioactive compounds using polymeric delivery systems, particularly poly (lactic-co-glycolic acid) (PLGA) nanoparticles. These nanoparticle systems have demonstrated potential biological activities, including cytotoxic, apoptotic, and anti-angiogenic effects in A2780 human ovarian cancer cells. However, PLGA-based nanoparticles are primarily designed for pharmaceutical applications and may present challenges for functional food development due to formulation complexity, production costs, and regulatory considerations [16]. In contrast, nanoemulsion systems formulated from food-grade ingredients offer several advantages, including ease of preparation, scalability, improved dispersion of bioactive compounds, and potential compatibility with food matrices. Despite the growing interest in kombucha-derived delivery systems, limited studies have systematically compared kombucha produced from different tea substrates and subsequently selected the most suitable formulation for development into a food-grade nanoemulsion system. Furthermore, information regarding the encapsulation efficiency, release behavior, and cellular uptake of kombucha tea nanoemulsion remains limited.

Therefore, the present study was designed to address the information of kombucha produced from green, oolong, and black tea by evaluating the fermentation characteristics, bioactive compound profiles, antioxidant activities, and cytotoxicity. Based on the obtained results, the most promising kombucha formulation was selected for the development of a food-grade nanoemulsion. The novelty of this study lies in the integrated approach of comparing different tea substrates, identifying the most suitable kombucha formulation, and subsequently developing and characterizing a kombucha nanoemulsion through the assessment of encapsulation efficiency, release behavior, and cellular uptake in multiple cell models. The objectives of this study were to: (i) produce and concentrate the fermentation of kombucha tea from green, oolong and black tea; (ii) determine the bioactive compounds and antioxidant activity in kombucha tea; (iii) formulate and characterize kombucha tea nanoemulsion; (iv) evaluate stability and intracellular localization in RAW 264.7 macrophages, A549 lung epithelial cells, and Caco-2 intestinal epithelial cells.

## 2. Materials and Methods

### 2.1. Fermentation of Kombucha Tea

Kombucha tea was fermented using tea leaves such as green tea, oolong tea and black tea. Dried tea leaf (1 g) was boiled in distilled water for 15 min and added 10% (*w*/*v*) of sucrose. After cool down of tea infusion, the tea infusion was inoculated with 10% (*v*/*v*) starter broth derived from a symbiotic culture of bacteria and yeast (SCOBY) obtained from Tea Gallery Group (Thailand) Co., Ltd., Chiang Mai, Thailand. The starter broth had been previously fermented for 15 days and was used without the addition of a cellulose pellicle. Separate starter broths corresponding to each tea type were used for the respective fermentation treatments. The fermentation was carried out at room temperature (28 ± 2 °C) for 15 days. Subsequently, the kinetics fermentation of kombucha tea was analyzed for its total count of bacteria and yeast, total acidity, pH, and total soluble solids (°Brix) at 0, 3, 6, 9, 12, and 15 days of fermentation [4].

The numbers of viable yeasts and acetic acid bacteria in kombucha tea were determined using a standard spread plate assay. Yeasts were enumerated on YM agar supplemented with chloramphenicol, while acetic acid bacteria were cultured on YPM agar containing amphotericin B (CAISSON, Smithfield, UT, USA). The plates were incubated at 30 °C for a minimum of 3 days before colony counting.

The pH of kombucha tea was determined using a digital pH meter (Denver Instruments, Bohemia, NY, USA). Total titratable acidity was measured by titration with 0.1 M NaOH and expressed as grams of acetic acid equivalents per liter of sample (g/L).

Total soluble solids (TSS) were measured using a refractometer (RHB-62ATC, JEDTO, Halden, Norway) and expressed as °Brix. Ethanol content was determined using an ebulliometer (Dujardin-Salleron, Arcueil, France).

### 2.2. Concentration of Kombucha Tea

After fermentation, kombucha tea (1 L) was filtered through a 0.22 µm membrane filter. The filtrate was concentrated using a rotary evaporator (Heidolph, Schwabach, Germany) at 50 °C under reduced pressure (50 mbar) and subsequently lyophilized using a freeze dryer (Labconco, Kansas, MO, USA) at −50 °C under a pressure of 0.300 ± 0.200 mbar. The yield of concentrated kombucha tea was calculated using the following equation: Yield (%) = (W_1_/V_1_) × 100, where W_1_ is the weight of the concentrated kombucha tea and V_1_ is the initial volume of kombucha tea. The concentrated kombucha tea powder was dissolved in sterile deionized water at a concentration of 200 mg/mL and stored at −20 °C until further analysis.

### 2.3. Bioactive Compounds Detection in Concentrated Kombucha Tea

The bioactive compounds including organic acids (glucuronic acid, gluconic acid, D-saccharic acid 1,4-lactone (DSL), ascorbic acid, acetic acid and succinic acid) and tea compounds (catechin, epigallocatechin gallate (EGCG), caffeine and theaflavin) in concentrated kombucha tea were determined by high performance liquid chromatography (HPLC). All standard compounds were purchased from Sigma-Aldrich (Darmstadt, Germany). The kombucha tea concentrated samples were filtered through a 0.45 µm sterile microfilter and 20 µL of the filtrate was injected into the HPLC system (Agilent technologies 1200 series, Santa Clara, CA, USA). The C-18 column (4.6 × 150 mm, 5 µm; GL Sciences, Tokyo, Japan) employing a UV detector (210 nm) was used for the analysis. Moreover, the HPLC system was controlled with a flow rate of 0.8 mL per minute and a running time of 40 min at 25 °C. Six organic acids in kombucha tea were separated by 20 mM KH_2_PO_4_ elution buffer at pH 2.4 and the standards of organic acids were used for comparison with con-centrated kombucha tea [4].

Tea-derived compounds were analyzed using an HPLC system equipped with a UV detector (Agilent technologies 1200 series, Santa Clara, CA, USA) set at 276 nm and an Agilent Eclipse XDB-C18 column (4.6 × 150 mm, 5 µm; Agilent technologies 1200 series, Santa Clara, CA, USA). The mobile phase consisted of deionized water (solvent A) and methanol (solvent B; RCI Labscan, Bangkok, Thailand). Separation was achieved using a gradient elution program as follows: 100% A at 0 min, 50% A/50% B at 15 min, and 100% B at 30 min. Chromatographic analysis was performed at a flow rate of 1.0 mL/min with a total run time of 30 min. The concentrations of catechin, epigallocatechin gallate (EGCG), caffeine, and theaflavin were determined using external standard calibration curves generated from the corresponding reference standards [17].

Peak integration was performed using Agilent ChemStation software version A.06.10. External calibration curves were established for each standard compound, and the concentrations of target analytes in the concentrated kombucha samples were determined from the corresponding calibration equations. The linear regression equation of glucuronic acid (y = 412.38x + 13.731), gluconic acid (y = 764.01x + 43.665), DSL (y = 609.93x + 9.0997), ascorbic acid (y = 12,669x + 18.74), acetic acid (y = 4843.5x + 12.711), succinic acid (y = 325.29x + 132.01), catechin (y = 11.179x − 19.556), EGCG (y = 56.496x + 2925.3), caffeine (y = 27.618x + 304.59) and theaflavin (y = 39.107x + 962.33) was obtained with a correlation coefficient (r^2^) of 0.998–0.999, indicating good linearity. Compounds were quantified in the concentrated kombucha samples and expressed as mg/g concentrated kombucha tea.

### 2.4. Antioxidant Activities

#### 2.4.1. DPPH Radical Scavenging Assay

The antioxidant activity of concentrated kombucha tea was determined by 2,2′-diphenyl-1-picrylhydrazyl (DPPH) assay [18]. The 0.1 mM DPPH solution dissolved 95% ethanol and concentrated kombucha tea with various concentrations (1–10 mg/mL) were prepared using 0.5 mL of each sample and 150 µL of DPPH solution and incubated for 20 min in the dark at room temperature. The absorbance was measured at 517 nm using a spectrophotometer (Thermo Scientific GENESYS 20, Leicestershire, UK). The radical scavenging activity was calculated according to the following equation:

Percentages of DPPH free radical scavenging activity (%) = [(A1 − A2)/A1] × 100

where A1 was the absorbance of the DPPH solution and A2 was the absorbance of the sample mixed with the DPPH solution. The concentrations providing 50% scavenging (IC_50_) were calculated from the graph plotted between the free radical scavenging percentages and the sample concentrations. The antioxidant activity of the kombucha tea was expressed by comparing the samples to standard compound (gallic acid) and was expressed as milligrams of gallic acid equivalent per gram of kombucha tea (mg GAE/g kombucha tea).

#### 2.4.2. ABTS Radical Scavenging Assay

The antioxidant activity of concentrated kombucha tea was evaluated using the ABTS assay [19]. A 7 mM solution of 2,2′-azinobis (3-ethylbenzothiazoline-6-sulphonic acid) (ABTS) was prepared in deionized water. The ABTS radical cation was generated by mixing the 7 mM ABTS solution with 2.45 mM potassium persulfate (K_2_S_2_O_8_) in a 1:1 ratio. The mixture was allowed to stand at room temperature for 12–16 h to form the radical solution. Prior to analysis, the ABTS working solution was diluted with deionized water to obtain an absorbance of 0.70 ± 0.002 at 734 nm. For the assay, 5 µL of concentrated kombucha tea with various concentrations (1–10 mg/mL) was added to 195 µL of the prepared ABTS reagent. After 10 min of incubation, the absorbance was measured at 734 nm. A standard curve was constructed using Trolox at concentrations ranging from 25 to 450 µg/mL, based on absorbance values at 734 nm. The radical scavenging activity was calculated according to the following equation:

Percentages of ABTS free radical scavenging activity (%) = [(A1 − A2)/A1] × 100

where A1 was the absorbance of the ABTS solution and A2 was the absorbance of the sample mixed with the ABTS solution. The concentrations providing 50% scavenging (IC_50_) were calculated from the graph plotted between the free radical scavenging percentages and the sample concentrations. The antioxidant activity of the kombucha tea was expressed by comparing the samples to standard compound (Trolox) and was expressed as milligrams of Trolox equivalent per gram of kombucha tea (mg TE/g kombucha tea).

#### 2.4.3. Ferric Reducing Antioxidant Power (FRAP) Assay

The antioxidant activity of concentrated kombucha tea was evaluated using the ferric reducing antioxidant power (FRAP) assay [20]. This method relies on the reduction of the ferric–TPTZ (2,4,6-tripyridyl-s-triazine) complex to its ferrous (Fe^2+^) form under acidic conditions. The FRAP reagent was freshly prepared by combining 300 mM acetate buffer (pH 3.6), 10 mM TPTZ dissolved in 40 mM HCl, and 20 mM FeCl_3_ in a ratio of 10:1:1. For the analysis, 50 µL of concentrated kombucha tea (0.2 and 2.0 mg/mL) was mixed with 100 µL of FRAP reagent. After incubation for 15 min, the absorbance was measured at 593 nm and quantified using a Trolox standard calibration curve prepared at concentrations ranging from 10 to 100 µg/mL. The antioxidant activity was expressed as milligrams of Trolox equivalent per gram of kombucha tea (mg TE/g kombucha tea).

### 2.5. Cytotoxicity

The cytotoxicity of concentrated kombucha tea was tested on human lung adenocarcinoma cells (A549 cells), human colorectal carcinoma cells (Caco-2) and murine macrophage cells (RAW 264.7 cells) using MTT assay [21]. RAW 264.7, Caco-2, and A549 cells were cultured in DMEM (Gibco, Grand Island, NY, USA) supplemented with 10% (*v*/*v*) heat-inactivated fetal bovine serum, 100 U/mL penicillin, and 100 µg/mL streptomycin. Cells were maintained at 37 °C in a humidified incubator with 5% CO_2_. Prior to experiments, the cells were washed twice with PBS (pH 7.4) and harvested using 0.05% (*v*/*v*) trypsin–EDTA solution. The cells (1 × 10^5^ cells/mL) were seeded into 96-well plates and incubated at 37 °C in a humidified atmosphere containing 5% CO_2_ for 24 h. After incubation, kombucha tea samples at concentrations ranging from 0.625 to 10 mg/mL were sterilized by filtration through a 0.22 µm membrane filter and subsequently added to the cells. Blank controls containing the corresponding concentrations of kombucha tea in culture medium without cells were included to determine for any interference in absorbance measurements. After 24 h of treatment, MTT solution (Bio Basic Inc., Amherst, NY, USA) was added to the cells, followed by incubation for an additional 4 h at 37 °C. The generated formazan crystals were solubilized with dimethyl sulfoxide (DMSO), and absorbance was recorded at 540 and 630 nm using a microplate reader (EZ Read 2000, Biochrom, Cambridge, UK). Cell viability (%) was calculated relative to the untreated control group.

### 2.6. Formulation of Concentrated Kombucha Tea Nanoemulsion

The nanoemulsion was formulated to encapsulate concentrated kombucha tea. The formulation consisted of propylene glycol (30%, *w*/*v*), vitamin E (10%, *w*/*v*), Tween 80 (10%, *w*/*v*), 200 mg/mL concentrated kombucha tea (12.5%, *v*/*v*), and deionized water (37.5%, *v*/*v*) to a final volume of 100%. Vitamin E served as the primary oil phase, while Tween 80 acted as the emulsifier and propylene glycol functioned as co-solvent [22]. All ingredients used in the formulation were food-grade materials. The oil phase, consisting of propylene glycol, vitamin E, Tween 80, and concentrated kombucha tea, was slowly added dropwise through a 29GX syringe (0.33 × 13 mm) into the aqueous phase under magnetic stirring (IKA, Staufen, Germany) at 1500 rpm and room temperature. High-shear homogenization or ultrasonication was not applied during the preparation process. The resulting nanoemulsion was stirred continuously for 30 min and stored at 4 °C until further analysis. The nanoemulsion was characterized for particle size, polydispersity index (PDI), and zeta potential using a Zetasizer (Malvern Instruments, Malvern, Worcs, UK). Particle morphology was examined by transmission electron microscopy (JEOL JEM-2100 Plus, Tokyo, Japan).

### 2.7. Encapsulation Efficiency

The encapsulated kombucha tea in nanoemulsion was investigated by an indirect quantification method using UV-Vis spectrophotometer [23]. Firstly, the kombucha tea was investigated the maximum absorption wavelength (λ_max_) by scanning over the range 200–800 nm by a UV spectrum. A standard calibration curve for kombucha tea was generated using various concentrations of kombucha tea (1.25–40 mg/mL). Second, the supernatant contained free kombucha tea was removed from kombucha tea nanoemulsion by centrifugation at 14,000 rpm, 30 min at 4 °C. In addition, the nanoemulsion particles were lysed by mixing with 10% sodium dodecyl sulfate and determined the absorbance. The absorbance of the nanoemulsion without kombucha tea was also measured and used as a background control. The background absorbance was subtracted before calculating the encapsulation efficiency (%). The amount of kombucha tea encapsulated in nanoemulsion was compared to standard curve of kombucha tea and calculated the percentage of encapsulation efficacy using the following equations:

Encapsulation Efficiency (%) = (amount of kombucha tea encapsulated in nanoemulsion/initial kombucha tea added) × 100

### 2.8. In Vitro Release Assay

The kombucha tea release from nanoemulsion was determined using a modified method [24]. The in vitro release profile of kombucha tea nanoemulsion was investigated by dispersing 2% (*v*/*v*) of the nanoemulsion in 50 mL of PBS (pH 7.0) and incubating the mixture at 37 °C with constant agitation. Samples were collected at designated time points (0, 10, 20, 30, 40, 50, 60, 120, and 180 min) and subsequently centrifuged at 14,000 rpm for 30 min at 4 °C. The resulting supernatants were used for further analysis. The supernatant was collected, and its absorbance was measured. The percentage of kombucha tea released from the nanoemulsion was then calculated using the kombucha tea standard calibration curve.

### 2.9. Intracellular Localization of Kombucha Tea Nanoemulsion into Cell Cultures

The human lung adenocarcinoma cells (A549 cells), human colorectal carcinoma cells (Caco-2) and murine macrophage cells (RAW 264.7 cells) at 50,000 cells/mL were plated to 96-well plate and incubated at 37◦C in a 5% CO_2_ incubator for 24 h. The kombucha tea nanoemulsion tagged with 20 µg/mL indocyanine green (ICG) was diluted with DMEM medium for 1000-fold before testing into the cell cultured [25]. After 2 h incubation, the particles were removed and washed with 1× PBS twice. The cells were fixed with 4% parafomadehyde for 10 min and incubated with 0.1% triton-x100 for 20 min at room temperature. The cells were washed with 1× PBS twice and stained with 1 µg/mL DAPI for 15 min. After washing with PBS, the cells were observed the kombucha tea nanoemulsion localization in the cells under fluorescence microscope (ECLIPSE Ts2R-FL, Nikon, Tokyo, Japan). The kombucha tea nanoemulsion exhibited green fluorescence from ICG, while the cell nuclei were stained blue with DAPI.

### 2.10. Storage Stability

The physical stability of the kombucha nanoemulsion was evaluated after storage at 4 °C for 6 months. Particle size, polydispersity index (PDI), and zeta potential were measured using a Zetasizer and compared with the freshly prepared nanoemulsion (Day 0). All measurements were performed in triplicate.

### 2.11. Statistical Analysis

All experiments were conducted independently on three separate occasions. Data are presented as mean ± standard deviation (SD) of three independent experiments (*n* = 3). Statistical analysis of characterization and stability data of kombucha tea nanoemulsion were performed using paired-sample *t*-tests. Data obtained from HPLC analysis and antioxidant activity assays were analyzed using one-way analysis of variance (ANOVA) followed by Tukey’s honestly significant difference (HSD) test for multiple comparisons. Statistical analyses were performed using IBM SPSS Statistics version 20. Differences were considered statistically significant at *p* < 0.05.

## 3. Results

### 3.1. Kinetics Fermentation of Kombucha Tea

**Figure 1:**
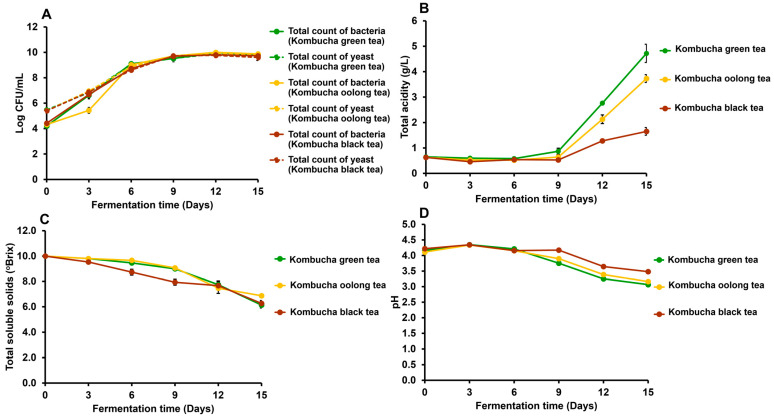
Fermentation of kombucha tea from green tea, oolong tea and black tea with bacteria and yeast for 15 days. The monitoring kinetics were observed total count of bacteria and yeast (**A**), total acidity (**B**), total soluble solids, °Brix (**C**) and pH (**D**). The results are presented as mean ± SD of three independent experiments.

The kombucha tea prepared from green tea, oolong tea and black tea were fermented with bacteria and yeast for 15 days. The populations of bacteria and yeast increased during fermentation. On day 12 of fermentation, all three types of kombucha showed the highest microbial growth, with bacterial counts reaching log 9.98 ± 0.12, log 10.00 ± 0.05, and log 9.81 ± 0.12 CFU/mL, and yeast counts reaching log 9.89 ± 0.08, log 9.93 ± 0.02, and log 9.77 ± 0.01 CFU/mL, respectively (Figure 1). Furthermore, on day 15 of fermentation, the pH of kombucha decreased while the total acidity increased. Kombucha produced from green tea exhibited the lowest pH value (3.06 ± 0.03) and the highest total acidity (4.72 ± 0.36 g/L) (Figure 1). Additionally, microorganisms utilized sugars during kombucha fermentation, as indicated by the °Brix values after 15 days of fermentation, which ranged from 6.1 ± 0.2 to 6.9 ± 0.1 °Brix (Figure 1).

### 3.2. Bioactive Compounds in Concentrated Kombucha Tea

Kombucha tea at 15 days of fermentation was concentrated using evaporator and freeze dried. The concentrated kombucha tea showed yellow to brown color and sticky. Moreover, yield of kombucha tea from green tea, oolong tea and black tea was 6.41%, 6.60% and 6.33%, respectively. In this study, the bioactive compounds in concentrated kombucha tea including organic acids (glucuronic acid, gluconic acid, DSL, ascorbic acid, acetic acid and succinic acid) and tea compounds (catechin, EGCG, caffeine and theaflavin) were determined by HPLC assay. The results demonstrated that black tea kombucha contained the highest EGCG content (13.225 ± 0.249 mg/g concentrated kombucha tea), followed by oolong tea (7.745 ± 0.048 mg/g) and green tea (6.358 ± 0.204 mg/g). In contrast, concentrated green tea kombucha exhibited higher levels of several other bioactive compounds, particularly gluconic acid, DSL, acetic acid, succinic acid, catechin, and caffeine, compared with oolong tea and black tea kombucha (Table 1 and Figures S1 and S2). Based on its overall bioactive compound profiles and antioxidant properties, green tea kombucha was selected for subsequent nanoemulsion development.

### 3.3. Antioxidant Activities of Concentrated Kombucha Tea

The antioxidant activity of concentrated kombucha prepared from green, oolong, and black teas was evaluated using the DPPH, ABTS, and FRAP assays. The results demonstrated that concentrated green tea kombucha exhibited the highest antioxidant properties among all samples. Specifically, it showed the strongest DPPH and ABTS radical scavenging activities, with values of 4.08 ± 0.41 mg GAE/g kombucha and 38.44 ± 1.51 mg TE/g kombucha, respectively. In addition, green tea kombucha exhibited the highest ferric reducing antioxidant power (FRAP), with a value of 64.34 ± 0.44 mg TE/g kombucha (Table 2).

### 3.4. Cytotoxicity of Concentrated Kombucha Tea on Cell Cultures

**Figure 2:**
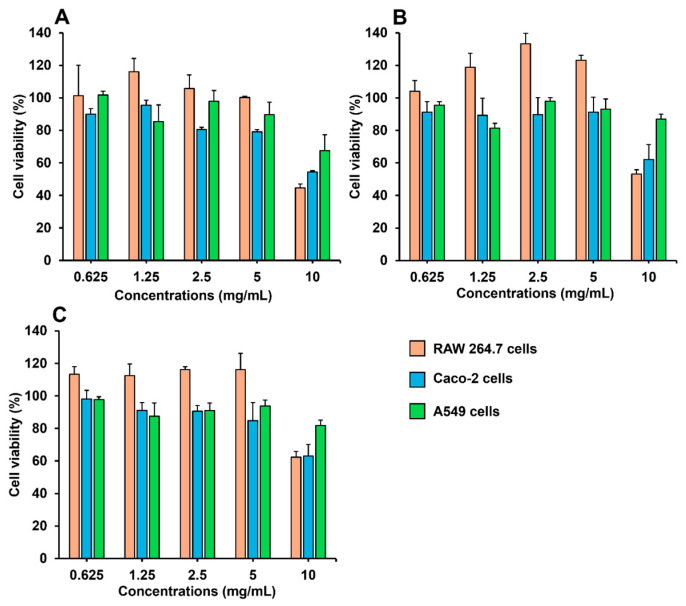
Cytotoxicity of concentrated kombucha from green tea (**A**), oolong tea (**B**), and black tea (**C**) on murine macrophage cells (RAW 264.7 cells), human colorectal carcinoma cells (Caco-2) and human lung adenocarcinoma cells (A549 cells). The data are presented as mean ± SD of triplicate independent experiments.

Concentrated kombucha tea was determined the toxicity on various cell cultures including murine macrophage cells (RAW 264.7 cells), human colorectal carcinoma cells (Caco-2) and human lung adenocarcinoma cells (A549 cells) using MTT assay. After 24 h treatment, the viability of all cells was presented more than 80% that revealed concentrated kombucha tea ranging from 0.625 to 5.0 mg/mL in all tested cells (Figure 2).

### 3.5. Concentrated Kombucha Green Tea Nanoemulsion

**Figure 3:**
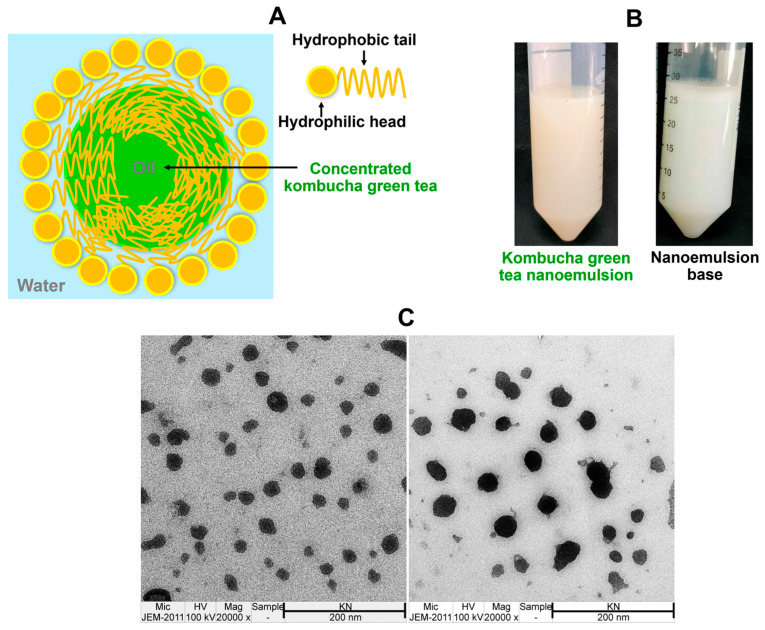
Characterization of concentrated green tea kombucha nanoemulsion. (**A**) Schematic representation of the nanoemulsion structure, illustrating the encapsulation of concentrated green tea kombucha within the oil phase stabilized by surfactant molecules. (**B**) Visual appearance of the developed nanoemulsion compared with the nanoemulsion base. (**C**) TEM images of the nanoemulsion, showing the particle size distribution and morphology of the nanoemulsion droplets.

Based on the evaluation of concentrated kombucha tea in terms of bioactive compound content, antioxidant activity, and cytotoxicity toward cultured cells, the concentrated green tea kombucha exhibited the highest efficacy. Therefore, it was selected for the development of nanoparticles in the form of a nanoemulsion (Figure 3A). The green tea extract at a concentration five times higher than the non-cytotoxic level (25 mg/mL) was used. The concentrated kombucha green tea was then incorporated into the oil phase consisting of 30% propylene glycol, 10% vitamin E, and 10% Tween 80, followed by gradual addition into the aqueous phase (deionized water) under continuous stirring using a magnetic stirrer (Figure 3B). The particle size of the concentrated green tea kombucha nanoemulsion was subsequently analyzed using a Nanosizer. The results showed that the nanoemulsion particles had an average size of 110.03 ± 6.2 nm. The polydispersity index (PDI) was 0.28 ± 0.01, indicating a moderately uniform size distribution. In addition, the zeta potential was +32.5 ± 0.5 mV, suggesting relatively good stability (Table 3). Moreover, the empty nanoemulsion exhibited a particle size of 104.34 ± 4.3 nm, a PDI of 0.24 ± 0.11, and a zeta potential of +30.8 ± 0.7 mV, which were comparable to those observed for the kombucha tea nanoemulsion (Table 3). The positive surface charge of the nanoparticles is advantageous as it helps reduce particle aggregation and enhances nanoparticle stability. Moreover, it improves cellular uptake and interaction with cell membranes, as most cells possess negatively charged surfaces composed of phospholipids and proteins. Transmission electron microscopy (TEM) analysis revealed that the nanoemulsion particles exhibited both spherical and oval morphologies (Figure 3C).

### 3.6. Encapsulation and Release Efficiency of Concentrated Green Tea Kombucha Nanoemulsion

**Figure 4:**
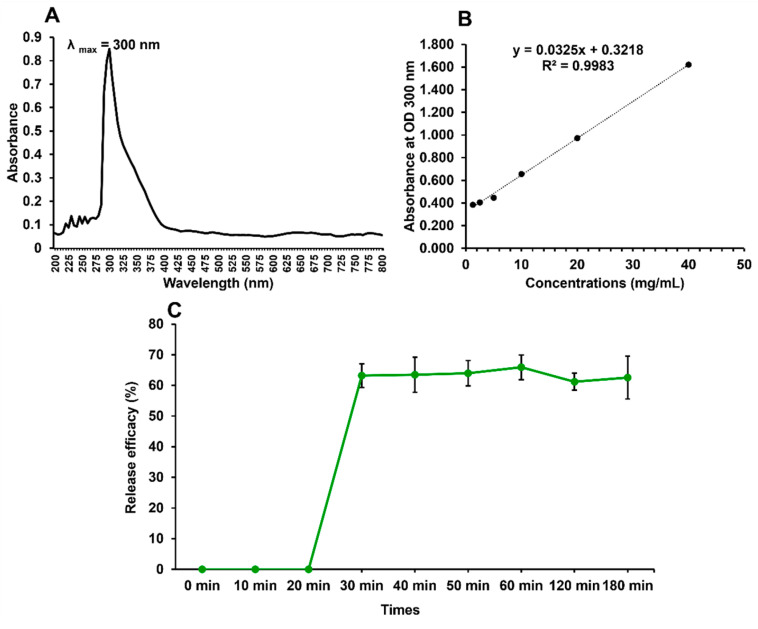
UV spectrum of concentrated green tea kombucha (**A**), standard calibration curve of concentrated green tea kombucha (**B**), and release profile of the concentrated green tea kombucha nanoemulsion under controlled conditions (pH 7.0, 37 °C) (**C**).

The encapsulation efficiency of the concentrated green tea kombucha nanoemulsion was evaluated by centrifugation to remove the unencapsulated fraction. The collected nanoemulsion particles were then disrupted using 10% SDS. Subsequently, the absorbance was measured at 300 nm, which corresponds to the maximum absorption wavelength (λ_max) of concentrated green tea kombucha, as shown in Figure 4A. The obtained absorbance values were compared with the standard calibration curve of concentrated green tea kombucha (Figure 4B) to calculate the encapsulation efficiency. The results demonstrated that the nanoemulsion formulation achieved a high encapsulation efficiency of 91.66 ± 2.29%. The release behavior of the concentrated green tea kombucha nanoemulsion was further investigated under controlled conditions (pH 7.0 and 37 °C). The release profile was monitored from 0 to 180 min. The results indicated that more than 60% of the encapsulated compounds were released within 30 min, followed by a sustained release pattern up to 2 h (Figure 4C).

### 3.7. Localization of Kombucha Green Tea Nanoemulsion into Intracellular Cell Cultures

**Figure 5:**
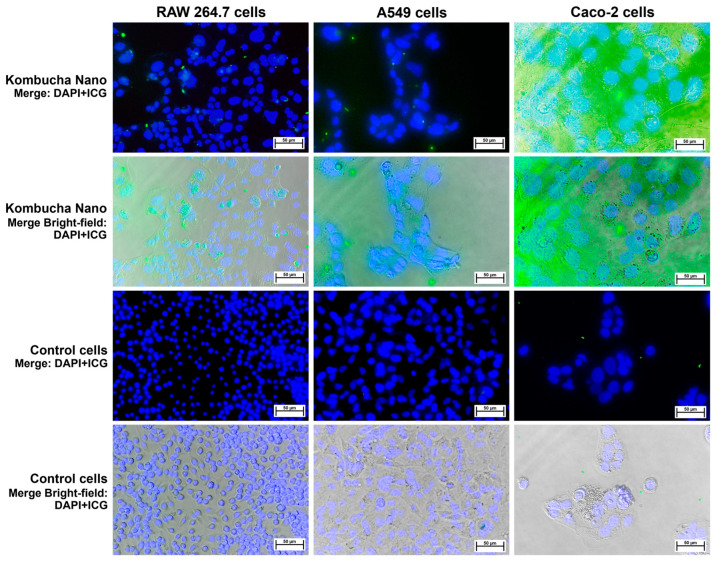
Cellular uptake of concentrated green tea kombucha nanoemulsion in RAW 264.7 macrophages, A549 lung epithelial cells, and Caco-2 intestinal epithelial cells, observed under a fluorescence microscope at 40× magnification. The nanoemulsion particles were labeled with indocyanine green (ICG), exhibiting green fluorescence, while cell nuclei were stained with DAPI, showing blue fluorescence.

The concentrated green tea kombucha nanoemulsion was labeled with the fluorescent dye indocyanine green (ICG) and subsequently evaluated for cellular uptake in RAW 264.7 macrophages, A549 lung epithelial cells, and Caco-2 intestinal epithelial cells at 2 h of incubation. Fluorescence microscopy revealed green fluorescence signals, indicating that the kombucha nanoemulsion was internalized by all cell types studied. In Caco-2 and A549 cells, the fluorescence appeared as small punctate signals distributed throughout the cytoplasm and in the perinuclear region, suggesting that cellular uptake of the nanoparticles likely occurred via endocytosis (Figure 5 and Figures S3–S5). In contrast, RAW 264.7 macrophages exhibited more intense and abundant fluorescence signals dispersed within the cytoplasm, consistent with their higher uptake capacity for nanoparticles (Figure S3). In all cell types, the fluorescence signals were predominantly localized in the cytoplasm, with minimal overlap with the nuclei stained with DAPI. This observation suggested that most of the nanoemulsion particles were retained within intracellular vesicles rather than directly entering the nucleus (Figure 5 and Figures S3–S5).

### 3.8. Storage Stability of Kombucha Tea Nanoemulsion

The physicochemical stability of the kombucha tea nanoemulsion was evaluated after storage at 4 °C for 6 months (Table 4). The particle size increased significantly (*p* < 0.05) from 110.03 ± 6.2 nm at Day 0 to 153.76 ± 9.4 nm after storage. However, the particle size remained within the nanoscale range (<200 nm). The polydispersity index (PDI) showed no significant change (*p* > 0.05), with values of 0.28 ± 0.01 and 0.24 ± 0.04 at Day 0 and after 6 months, respectively. In contrast, the zeta potential decreased significantly (*p* < 0.05) from +32.5 ± 0.5 mV to +28.7 ± 0.9 mV during storage. Despite these changes, the nanoemulsion maintained a low PDI and a zeta potential, indicating acceptable physicochemical stability under refrigerated storage conditions.

## 4. Discussion

The present study demonstrates that kombucha fermentation significantly affects microbial dynamics, physicochemical properties, and bioactive compound profiles depending on the type of tea substrate. The observed increase in microbial populations, particularly the peak at day 12, is consistent with the symbiotic metabolism of yeasts and acetic acid bacteria, which convert sugar into ethanol and subsequently into organic acids such as acetic, gluconic, and glucuronic acids [26]. The decrease in pH and increase in total acidity, particularly in green tea kombucha, are consistent with previous reports showing that organic acid accumulation intensifies during the later stages of fermentation (12–15 days) [4,27].

The differences observed among tea types can be explained by their phytochemical compositions. Green tea is known to contain higher levels of catechins, particularly EGCG, compared to oolong and black tea, which undergo partial or full oxidation during processing [28]. During fermentation, microbial enzymes modify these compounds, leading to both degradation and biotransformation of catechins into simpler phenolics or derivatives with enhanced bioactivity [29]. These evidences support the findings of the present study since concentrated green tea kombucha exhibited higher levels of bioactive compounds, including catechin, caffeine and organic acids (gluconic acid, DSL, acetic acid and succinic acid) whereas black tea kombucha exhibited the highest epigallocatechin gallate (EGCG) concentration. Although several major organic acids and tea-derived bioactive compounds were quantified in this study, the metabolic transformations occurring during kombucha fermentation were considerably more complex. Future studies employing comprehensive metabolomic approaches could provide deeper insights into fermentation-derived metabolites and the biotransformation pathways of tea polyphenols.

The superior antioxidant activity observed in green tea kombucha is consistent with previous studies demonstrating a strong correlation between phenolic content and antioxidant activity [30]. Fermentation has been shown to enhance antioxidant potential by releasing bound phenolics and generating new metabolites with improved radical scavenging ability [29]. Additionally, the presence of organic acids and other metabolites may contribute synergistically to the overall antioxidant activity. EGCG is a powerful antioxidant polyphenol, most abundant in green tea, known for neutralizing cellular damage from free radicals. It promotes health by reducing inflammation, aiding in weight loss, and supporting cardiovascular, neural, and skin health, with potential anti-cancer properties [31]. Moreover, catechins which are dominant in green tea, neutralize free radicals and chelate metals, while caffeine stimulates the central nervous system. Together, they offer anti-inflammatory, antimicrobial, and anti-aging benefits, with green tea providing higher catechin levels compared to black tea. Green tea kombucha exhibited the highest antioxidant performance among the tested samples in all three assays.

The FRAP assay primarily measures electron transfer activity, reflecting the reducing power of bioactive compounds such as catechins and EGCG, which are abundant in green tea [32]. Previous studies have reported that fermentation significantly enhances FRAP values due to the increased availability of reducing compounds [33]. In contrast, the ABTS assay evaluates both electron transfer and hydrogen atom transfer mechanisms, resulting in slightly lower but still significant antioxidant values. Meanwhile, the DPPH assay is more sensitive to steric hindrance and is less suitable for aqueous systems, which may explain its lower values in kombucha samples. Furthermore, the antioxidant activity of kombucha has been strongly correlated with its total phenolic content, indicating that tea-derived polyphenols are the primary contributors to its antioxidant activity, while fermentation-derived metabolites may play a supportive role [29].

The low cytotoxicity observed across RAW 264.7, Caco-2, and A549 cell lines suggests that concentrated kombucha is biocompatible at the tested concentrations. This aligns with previous reports highlighting the safety and potential health benefits of tea-derived polyphenols, which exhibit antioxidant, anti-inflammatory, and anticancer properties [34]. These findings highlight the potential of kombucha as a promising functional food ingredient, owing to its rich content of bioactive compounds and strong antioxidant activity. In food applications, the stability and bioavailability of these compounds are critical factors affecting their efficacy. Therefore, a nanoemulsion-based delivery system was developed to enhance the physicochemical stability, protect sensitive compounds from degradation, and facilitate their incorporation into food matrices. Such systems may improve the functional performance of kombucha-derived ingredients in food and nutraceutical formulations. In addition, nanoemulsion systems are widely recognized in food science for their ability to improve the dispersibility of bioactive compounds and protect them from environmental degradation. These properties may contribute to improved stability and delivery performance in food applications. This highlights the potential of the developed kombucha nanoemulsion for incorporation into beverages, functional drinks, and nutraceutical formulations [35,36].

The resulting nanoemulsion exhibited a particle size of approximately 110 nm, low PDI, and high positive zeta potential, indicating good stability. The formulation was designed using food-grade ingredients commonly employed in nanoemulsion systems for food applications. Tween 80 was selected as a nonionic surfactant due to its high emulsification efficiency and ability to reduce interfacial tension between oil and aqueous phases [37]. Propylene glycol acted as a co-solvent that facilitated the dispersion of kombucha-derived compounds within the formulation, while vitamin E served as the oil phase and contributed to the formation of stable nano-sized droplets [38,39]. The combination of these components resulted in a nanoemulsion with a small particle size, narrow size distribution, and high encapsulation efficiency, demonstrating its suitability as a carrier system for kombucha-derived bioactive compounds [40]. Similar findings have been reported in nanoemulsion systems containing tea polyphenols, where nanoscale size has been associated with improved solubility, physicochemical stability, and cellular interaction of encapsulated bioactive compounds [41]. The high encapsulation efficiency (~92%) observed in this study further supports the effectiveness of nanoemulsions in protecting sensitive phytochemicals from degradation. Previous research has demonstrated the feasibility of developing nano-scale delivery systems from kombucha-derived materials. For example, bacterial nanocellulose produced during kombucha fermentation has been successfully utilized as a carrier for bioactive compounds and probiotics due to its high surface area and biocompatibility [42]. In addition, microencapsulation approaches have been applied to preserve kombucha bioactivity and improve stability. Furthermore, studies incorporating kombucha-derived materials into nano-structured systems, such as nanocomposite hydrogel and nanoemulsion, have shown promising physicochemical properties and functional performance [43]. These findings support the potential of kombucha as a novel source for nano-delivery systems. Therefore, the development of a kombucha-based nanoemulsion in the present study represents an important advancement in this field.

Importantly, to the best of our knowledge, studies on nanoemulsion systems directly derived from kombucha are still limited, particularly those evaluating both physicochemical properties and cellular uptake. Therefore, the present study provides a novel contribution by integrating fermentation-derived bioactive compounds with nanoemulsion-based delivery for food applications.

The release profile demonstrated an initial burst release followed by sustained release, which is characteristic of nano-delivery systems. This biphasic release behavior may be advantageous by providing an initial rapid release followed by a more sustained release of encapsulated compounds. Previous studies have shown that nano-carriers can modulate release kinetics and influence the delivery behavior of encapsulated bioactive compounds [44]. Cellular uptake studies revealed that the nanoemulsion was efficiently internalized by all tested cell lines, with higher uptake observed in RAW 264.7 macrophages. This is consistent with the phagocytic nature of macrophages and their enhanced ability to internalize nanoparticles. In epithelial cells (Caco-2 and A549), the punctate fluorescence pattern suggests uptake via endocytosis, which has also been reported for catechin-loaded nanocarriers [45]. The localization of nanoparticles was primarily in the cytoplasm, rather than the nucleus, suggested intracellular vesicular trafficking and indicated that the nanoemulsion was successfully internalized by the cells.

The storage stability study demonstrated that the kombucha tea nanoemulsion maintained acceptable physicochemical stability during 6 months of refrigerated storage. Although the particle size increased significantly over time, it remained below 200 nm, indicating that the formulation retained its nanoscale characteristics. The slight reduction in zeta potential may be attributed to gradual changes at the droplet interface during storage. The zeta potential remained above +25 mV, which is generally considered sufficient to maintain colloidal stability [46]. Furthermore, the absence of a significant increase in PDI suggested that extensive droplet aggregation did not occur during storage. These findings support the suitability of the developed nanoemulsion for refrigerated storage and potential application in functional food systems. Although the nanoemulsion exhibited acceptable physicochemical stability during refrigerated storage, the present study did not directly compare the stability of encapsulated and non-encapsulated kombucha bioactive compounds. Therefore, the protective effect of nanoencapsulation against degradation of compounds could not be confirmed. Future studies should investigate the storage stability, oxidative stability, and degradation kinetics of individual bioactive compounds in both encapsulated and non-encapsulated systems.

However, this study has some limitations. The release behavior was evaluated under simplified *in vitro* conditions, which may not fully represent the complexity of gastrointestinal environments. Although the nanoemulsion exhibited acceptable physicochemical stability during six months of refrigerated storage, its stability under different storage temperatures and food processing conditions remains to be investigated. Furthermore, cytotoxicity assessments were performed only on concentrated kombucha extracts, while the cytotoxicity of the nanoemulsion formulation and blank nanoemulsion were not evaluated. The cellular uptake study did not include quantitative uptake analysis or normal epithelial cell models. However, future studies on *in vitro* digestion models, shelf-life stability, comprehensive safety evaluations, quantitative cellular uptake analysis, and application in real food matrices should be performed.

Overall, this study demonstrates that green tea kombucha is a rich source of bioactive compounds and that nanoemulsion-based delivery systems significantly improve their stability, bioavailability, and cellular uptake. These findings are consistent with previous research and further highlight the potential of combining fermentation and nanotechnology for developing of advanced functional food and therapeutic formulations. This approach provides a promising strategy for developing value-added fermented beverages, functional drinks, and innovative food products with enhanced health benefits and improved bioactive delivery efficiency.

## 5. Conclusions

In conclusion, this study demonstrated that the type of tea substrate significantly influences the fermentation characteristics, bioactive compound composition, and antioxidant activity of kombucha. Among the tested samples, green tea kombucha exhibited superior functional properties, including higher levels of catechins and organic acids, stronger antioxidant activity, and low cytotoxicity, supporting its potential as a functional food ingredient. The development of a nanoemulsion system further enhanced the applicability of concentrated green tea kombucha by providing a nano-sized formulation with high encapsulation efficiency, acceptable physicochemical stability, and controlled release behavior. The nanoemulsion also maintained acceptable physicochemical stability during 6 months of refrigerated storage, remaining within the nanoscale range despite moderate changes in particle size and zeta potential. In addition, the nanoemulsion showed effective cellular uptake, indicating its potential as a carrier for the intracellular delivery of encapsulated bioactive compounds. Importantly, this study demonstrates the feasibility of integrating fermentation-derived bioactive compounds with nanoemulsion-based delivery systems for food applications. The developed system may facilitate the incorporation of kombucha-derived ingredients into food matrices, such as functional beverages and nutraceutical products, while maintaining their physicochemical properties and delivery potential. Overall, this work provides a novel approach for enhancing the value of kombucha through nanotechnology and offers significant potential for the development of innovative functional food products. Future studies should focus on *in vitro* digestion, evaluation under different storage conditions, and application in real food systems to support industrial-scale utilization.

## Figures and Tables

**Table 1:** The contents of bioactive compounds in concentrated kombucha tea.

Compounds	Contents (mg/g Concentrated Kombucha Tea)		
	Kombucha Green Tea	Kombucha Oolong Tea	Kombucha Black Tea
**Organic acids**			
Glucuronic acid	0.189 ± 0.004 ^b^	0.127 ± 0.034 ^c^	0.216 ± 0.045 ^a^
Gluconic acid	3.685 ± 0.015 ^a^	2.444 ± 0.018 ^b^	1.309 ± 0.185 ^c^
DSL	0.201 ± 0.013 ^a^	0.137 ± 0.002 ^b^	0.030 ± 0.002 ^c^
Ascorbic acid	0.336 ± 0.00 ^b^	0.343 ± 0.001 ^a^	0.059 ± 0.014 ^c^
Acetic acid	3.700 ± 0.039 ^a^	3.277 ± 0.013 ^b^	1.136 ± 0.056 ^c^
Succinic acid	0.507 ± 0.134 ^a^	0.625 ± 0.138 ^a^	0.400 ± 0.029 ^b^
**Tea bioactive compounds**			
Catechin	5.179 ± 0.897 ^a^	2.842 ± 0.040 ^b^	ND ^c^
EGCG	6.358 ± 0.204 ^c,^*	7.745 ± 0.048 ^b,^*	13.225 ± 0.249 ^a,^*
Caffeine	5.286 ± 0.573 ^a^	2.009 ± 0.441 ^b^	ND ^c^
Theaflavin	ND ^a^	ND ^a^	ND ^a^

The data of different superscript letters (a, b, c) are presented as mean ± SD of triplicate independent experiments and show significantly different values within each concentrated kombucha tea (*p* < 0.05). * Values indicate the highest contents, which are significantly different (*p* < 0.05). ND: Not detected.

**Table 2:** Antioxidant activity of concentrated kombucha tea by DPPH, ABTS and FRAP assay.

Concentrated Kombucha Tea	DPPH		ABTS		FRAP
	IC_50_ (mg/mL)	Antioxidant Activity (mg GAE/g Kombucha)	IC_50_ (mg/mL)	Antioxidant Activity (mg TE/g Kombucha)	Antioxidant Activity (mg TE/g Kombucha)
Green tea	0.98 ± 0.19 ^a^	4.08 ± 0.41 ^a,^*	4.53 ± 0.18 ^a^	38.44 ± 1.51 ^a,^*	64.34 ± 0.44 ^a,^*
Oolong tea	1.40 ± 0.60 ^b^	3.05 ± 1.09 ^b^	5.54 ± 0.11 ^b^	31.41 ± 0.61 ^b^	45.05 ± 6.33 ^b^
Black tea	2.75 ± 0.83 ^c^	1.46 ± 0.32 ^c^	18.11 ± 2.22 ^c^	9.74 ± 1.25 ^c^	5.15 ± 0.16 ^c^

The data of different superscript letters (a, b, c) are presented as mean ± SD of triplicate independent experiments and show significantly different values within each concentrated kombucha tea (*p* < 0.05). * Values indicate the highest contents, which are significantly different (*p* < 0.05).

**Table 3:** Nanoparticle sizes, polydispersity index (PDI) and zeta potential values of concentrated kombucha green tea nanoemulsion and empty nanoemulsion.

Nanoparticles	Size (nm)	Polydispersity Index (PDI)	Zeta Potential (mV)
Concentrated kombucha green tea nanoemulsion	110.03 ± 6.2 ^a^	0.28 ± 0.01 ^a^	+32.5 ± 0.5 ^a^
Empty nanoemulsion	104.34 ± 4.3 ^b^	0.24 ± 0.11 ^a^	+30.8 ± 0.7 ^b^

The data of different superscript letters (a, b) are presented as mean ± SD of triplicate independent experiments and show significantly different values within concentrated kombucha green tea nanoemulsion and empty nanoemulsion (*p* < 0.05).

**Table 4:** Physicochemical stability of kombucha tea nanoemulsion during storage at 4 °C for 6 months.

Parameter	Day 0	6 Months
Size (nm)	110.03 ± 6.2 ^a^	153.76 ± 9.4 ^b^
PDI	0.28 ± 0.01 ^a^	0.24 ± 0.04 ^a^
Zeta potential (mV)	+32.5 ± 0.5 ^a^	+28.7 ± 0.9 ^b^

The data of different superscript letters (a, b) are presented as mean ± SD of triplicate independent experiments and show significantly different values between day 0 and 6 months (*p* < 0.05).

## Data Availability

The original contributions presented in this study are included in the article/supplementary material. Further inquiries can be directed to the corresponding author.

## Supplementary Materials

The following supporting information can be downloaded at: https://www.mdpi.com/article/10.3390/foods15142468/s1, Figure S1: Representative HPLC chromatograms of organic acid standards (glucuronic acid, gluconic acid, DSL, ascorbic acid, acetic acid and succinic acid) and concentrated kombucha samples prepared from green tea, oolong tea, and black tea. Figure S2: Representative HPLC chromatograms of tea compound standards (catechin, EGCG, caffeine and theaflavin) and concentrated kombucha samples prepared from green tea, oolong tea, and black tea. Figure S3: Cellular uptake of concentrated green tea kombucha nanoemulsion in RAW 264.7 macrophages, observed under a fluorescence microscope at 40× magnification. This image combines bright-field microscopy with fluorescence. Figure S4: Cellular uptake of concentrated green tea kombucha nanoemulsion in A549 lung epithelial cells, observed under a fluorescence microscope at 40× magnification. This image combines bright-field microscopy with fluorescence. Figure S5: Cellular uptake of concentrated green tea kombucha nanoemulsion in Caco-2 intestinal epithelial cells, observed under a fluorescence microscope at 40× magnification. This image combines bright-field microscopy with fluorescence.
