# Supplementary material for: Development and Characterization of Kombucha Tea Nanoemulsion for Stability, Bioactive Delivery, and Functional Food Applications
Source: Foods. 2026 Jul 12;15(14):2468. doi: 10.3390/foods15142468 (PMC13408310; doi:10.3390/foods15142468)
Supplement: Supplementary file 1 [file foods-15-02468-s001.zip › foods-4351392-supplementary.pdf]

Supplementary Materials

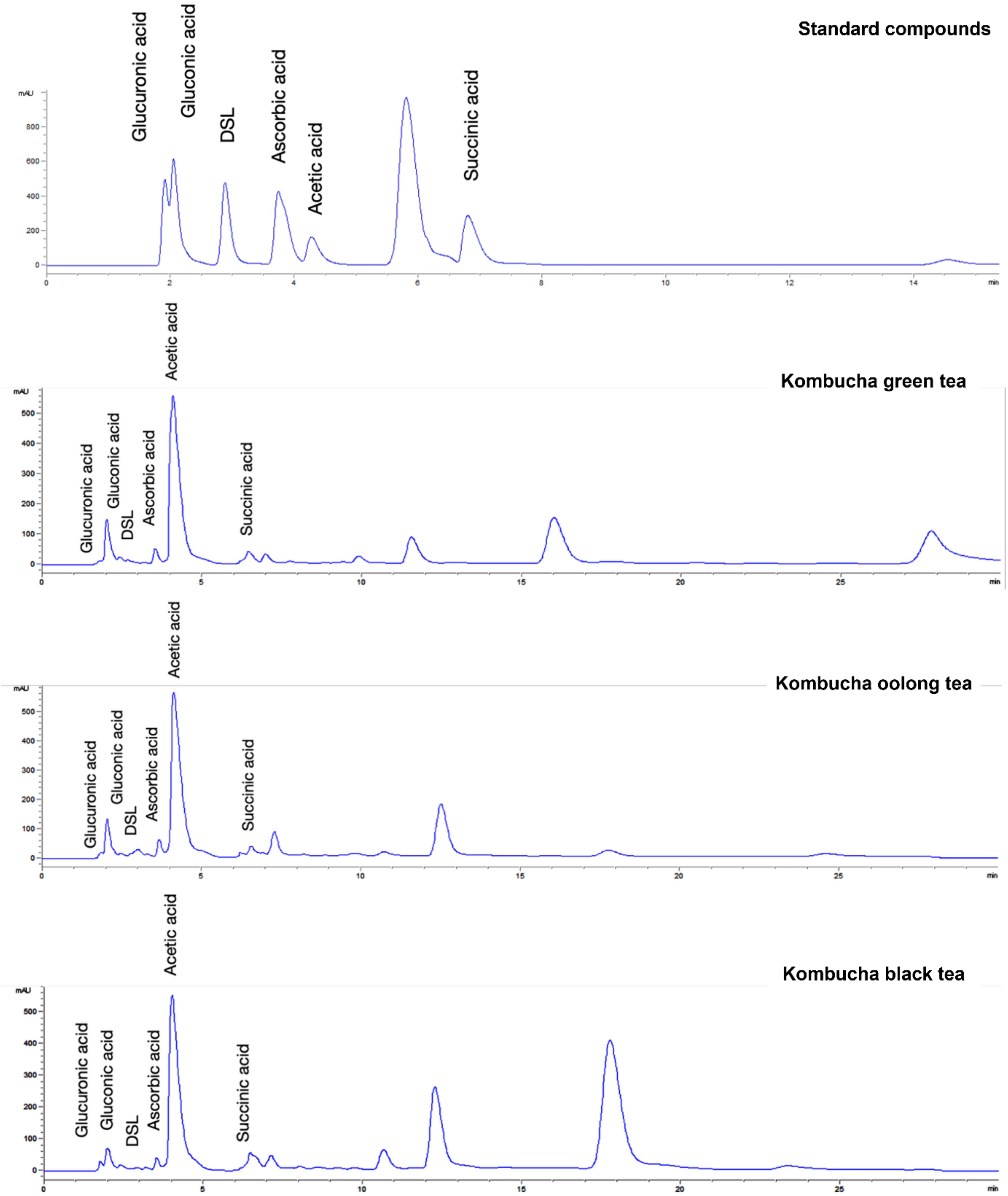

Figure S1. Representative HPLC chromatograms of organic acid standards (glucuronic acid, gluconic acid, DSL, ascorbic acid, acetic acid and succinic acid) and concentrated kombucha samples prepared from green tea, oolong tea, and black tea.

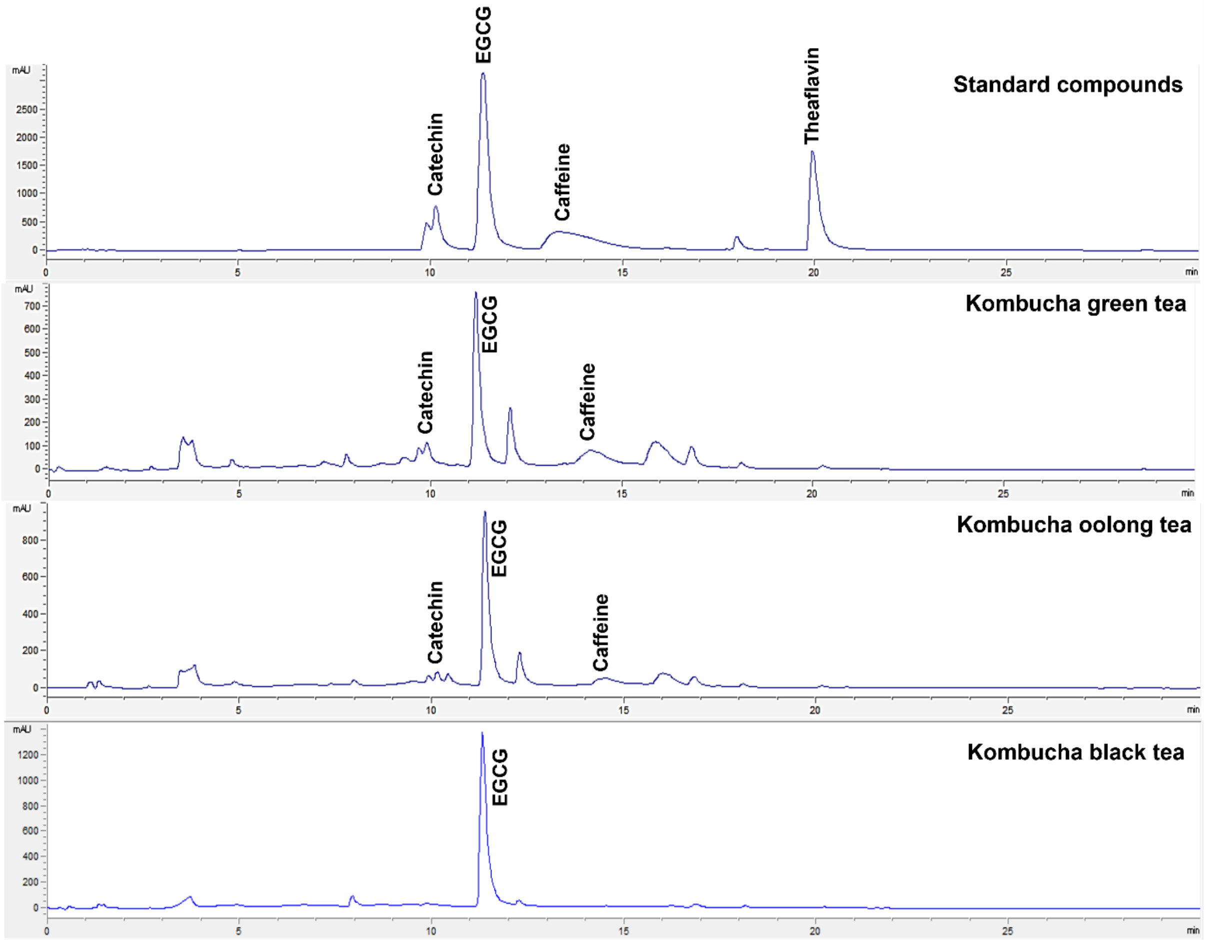

Figure S2. Representative HPLC chromatograms of tea compound standards (catechin, EGCG, caffeine and theaflavin) and concentrated kombucha samples prepared from green tea, oolong tea, and black tea.

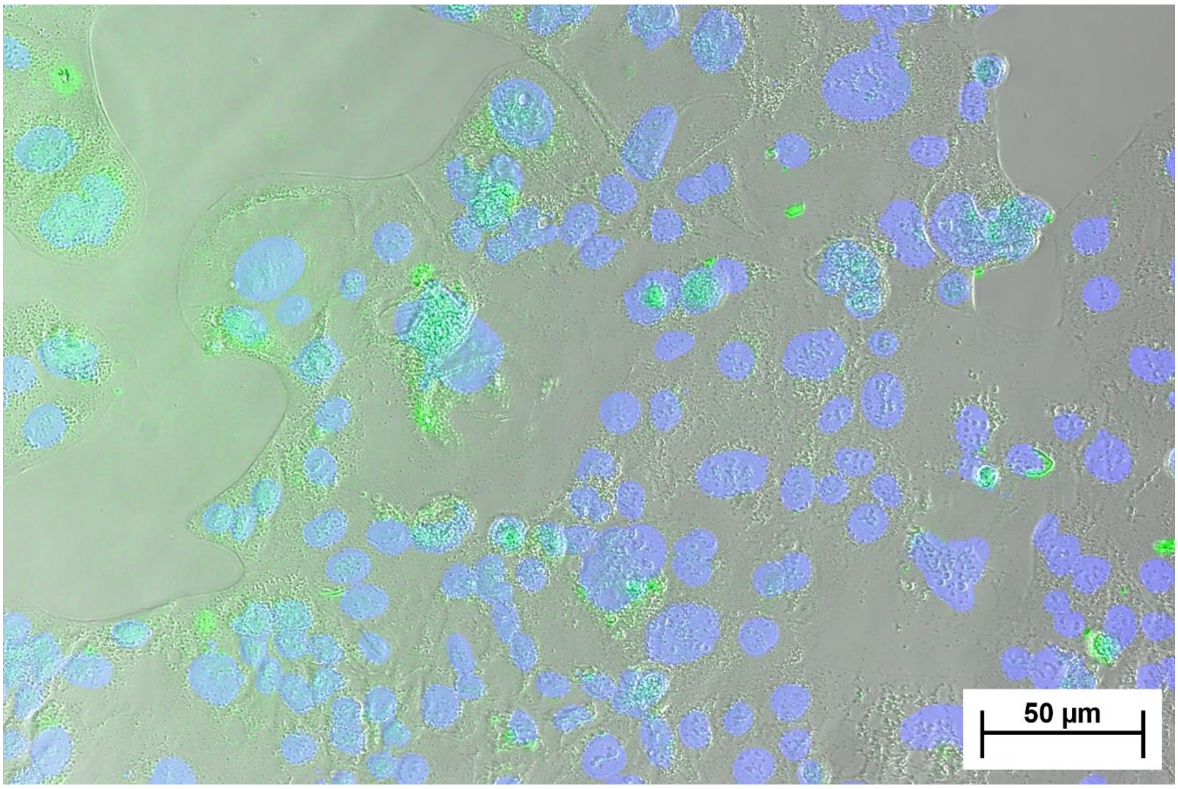

Figure S3. Cellular uptake of concentrated green tea kombucha nanoemulsion in RAW 264.7 macrophages, observed under a fluorescence microscope at 40X magnification. This image combines bright-field microscopy with fluorescence. The nanoemulsion particles were labeled with indocyanine green (ICG), exhibiting green fluorescence, while cell nuclei were stained with DAPI, showing blue fluorescence.

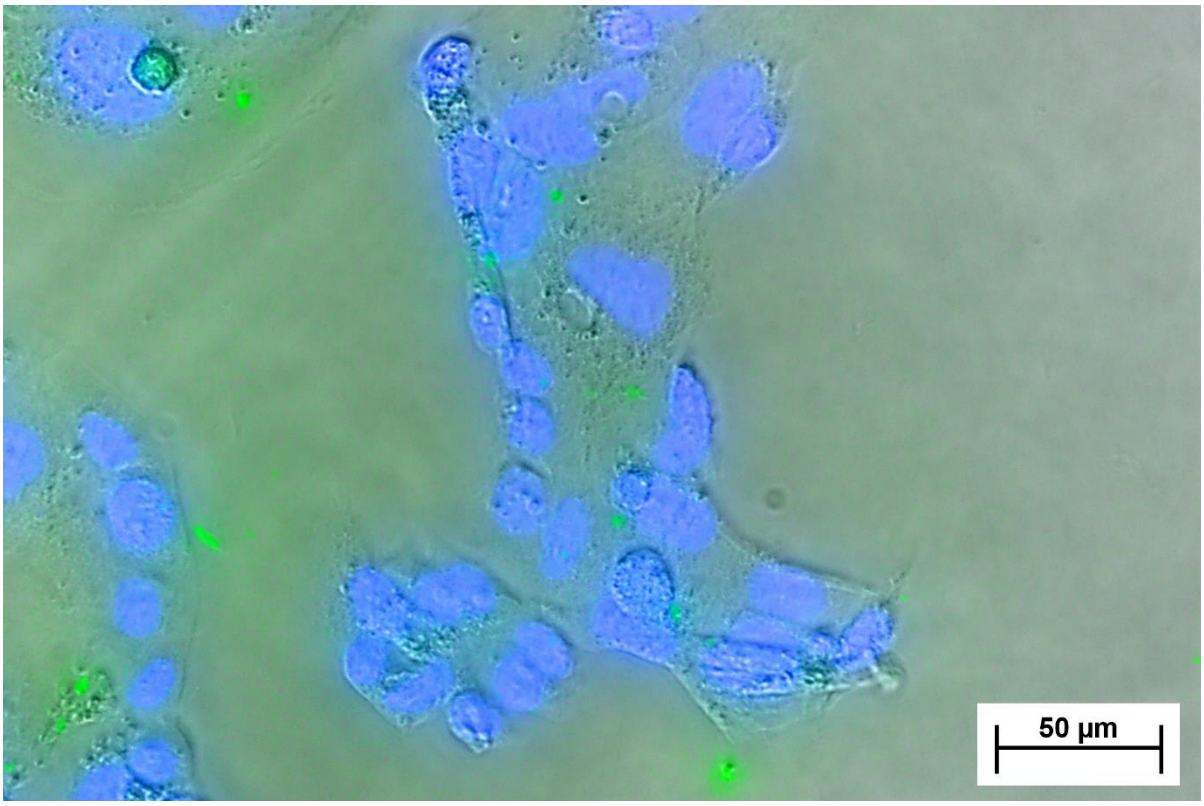

Figure S4. Cellular uptake of concentrated green tea kombucha nanoemulsion in A549 lung epithelial cells, observed under a fluorescence microscope at 40X magnification. This image combines bright-field microscopy with fluorescence. The nanoemulsion particles were labeled with indocyanine green (ICG), exhibiting green fluorescence, while cell nuclei were stained with DAPI, showing blue fluorescence.

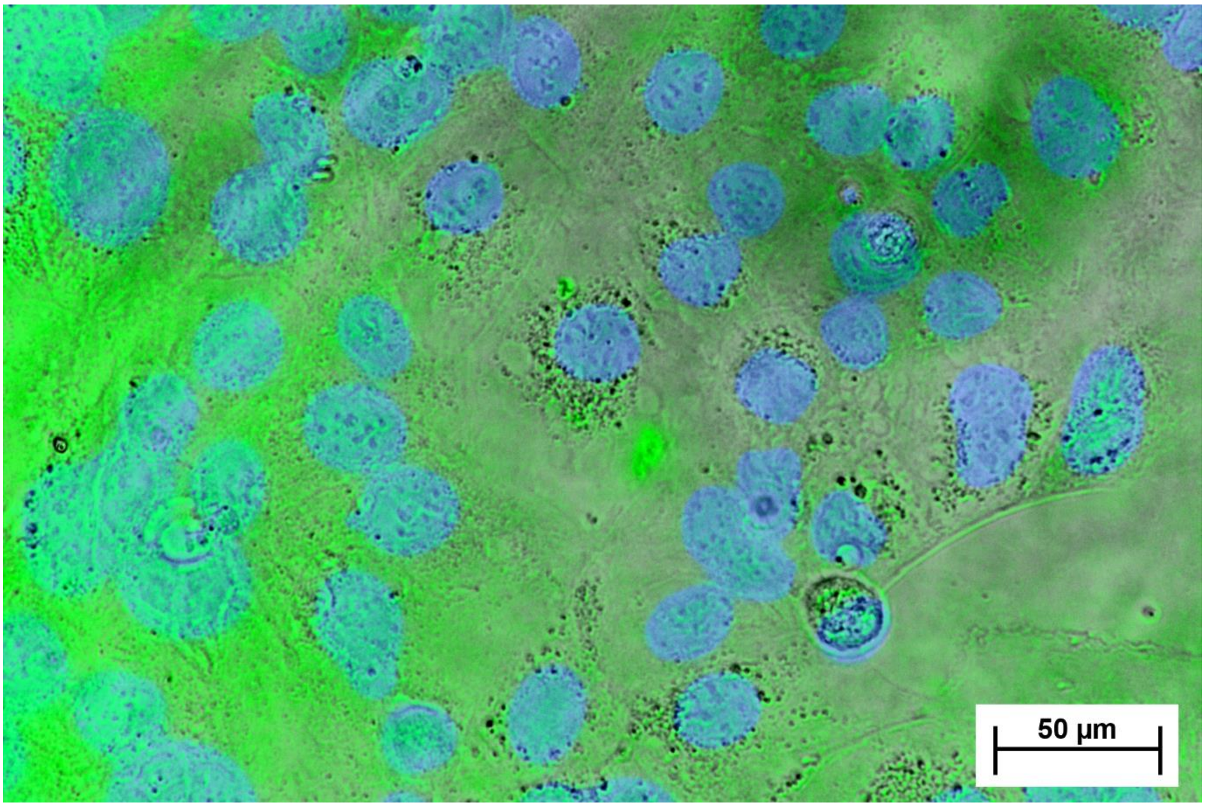

Figure S5. Cellular uptake of concentrated green tea kombucha nanoemulsion in Caco-2 intestinal epithelial cells, observed under a fluorescence microscope at 40X magnification. This image combines bright-field microscopy with fluorescence. The nanoemulsion particles were labeled with indocyanine green (ICG), exhibiting green fluorescence, while cell nuclei were stained with DAPI, showing blue fluorescence.
